# Comparative proteomic analysis of seminal plasma exosomes in buffalo with high and low sperm motility

**DOI:** 10.1186/s12864-022-09106-2

**Published:** 2023-01-09

**Authors:** Kai Yu, Kai Xiao, Qin-qiang Sun, Run-feng Liu, Liang-feng Huang, Peng-fei Zhang, Hui-yan Xu, Yang-qing Lu, Qiang Fu

**Affiliations:** 1 State Key Laboratory for Conservation and Utilization of Subtropical Agro-Bioresources, Guangxi University, Nanning, 530004 China; 2 College of Animal Science and Technology, Guangxi University, Nanning, 530004 China

**Keywords:** Buffalo, Seminal plasma, Exosomes, Proteome, Activity, Sperm motility

## Abstract

**Background:**

Exosomes are nanosized membranous vesicles secreted by various types of cells, which facilitate intercellular communication by transporting bioactive compounds. Exosomes are abundant in biological fluids including semen, and their protein composition and the potential of seminal plasma exosomes (SPEs) as fertility biomarkers were elucidated in humans, however, little information is available regarding buffalo (*Bubalus bubalis*). Here, we examined protein correlation between spermatozoa, seminal plasma (SP), and SPEs, and we compared and analyzed protein differences between high-motility (H-motility) and low-motility (L-motility) SPEs in buffalo.

**Results:**

SPEs were concentrated and purified by ultracentrifugation combined with sucrose density gradient centrifugation, followed by verification using western blotting, nanoparticle tracking analysis, and transmission electron microscopy. Protein composition in spermatozoa, SP and SPEs, and protein difference in H- and L-motility SPEs were identified by LC-MS/MS proteomic analysis and were functionally analyzed through comprehensive bioinformatics. Many SPEs proteins originated from spermatozoa and SP, and nearly one third were also present in spermatozoa and SP. A series of proteins associated with reproductive processes including sperm capacitation, spermatid differentiation, fertilization, sperm-egg recognition, membrane fusion, and acrosome reaction were integrated in a functional network. Comparative proteomic analyses showed 119 down-regulated and 41 up-regulated proteins in L-motility SPEs, compared with H-motility SPEs. Gene Ontology (GO) enrichment of differentially expressed proteins (DEPs) showed that most differential proteins were located in sperm and vesicles, with activities of hydrolase and metalloproteinase, and were involved in sperm-egg recognition, fertilization, single fertilization, and sperm-zona pellucida binding processes, etc. Kyoto Encyclopedia of Genes and Genomes (KEGG) analysis showed that differential proteins were mainly involved in the PPRP signaling pathway, calcium signaling pathway, and cAMP signaling pathway, among others. Furthermore, 6 proteins associated with reproduction were validated by parallel reaction monitoring analysis.

**Conclusion:**

This study provides a comprehensive description of the seminal plasma exosome proteome and may be of use for further screening of biomarkers associated with male infertility.

**Supplementary Information:**

The online version contains supplementary material available at 10.1186/s12864-022-09106-2.

## Background

Extracellular vesicles (EVs) are biological phospholipid bilayer vesicles secreted into the extracellular space by various cells. EVs are differentiated into three types, including microvesicles, apoptotic bodies, and exosomes [1, 2]. Exosomes are nanosized (30–150 nm diameter) spherical vesicles, occurring at a density ranging from 1.13 to 1.19 g/mL in sucrose density gradient solution. By contrast to other EVs, exosomes contain nucleic acids, proteins, lipids, transcription factor receptors, and other cytokines [1]. Exosomes can deliver bioactive substances and easily degradable compounds to target cells to elicit physiological and pathological processes. Among them, exosomal proteins play essential roles in cell communication [3]. Of note, the protein content of exosomes varies considerably between different cell or tissue sources.

The seminal plasma (SP) of mammals is a complex secretion produced by testicular tissue, accessory sex glands, and the epididymis, and it may affect sperm fertility [4, 5]. During the migration of spermatozoa to the epididymis, most proteins bind to the sperm membrane through exosomes. SP is considered to play key roles not only in activating immune mechanisms for protecting spermatozoa against oxidative stress [6], but also in regarding sperm physiology [7], morphology [8], and motility acquisition [9–12]. Considering that spermatozoa are transcriptionally silent cells, the acquisition of secretory products of SP is an intrinsic way to complete post-testicular maturation. Therefore, compounds encapsulated in seminal plasma exosomes (SPEs) and exosomal shuttle mechanisms with respect to intercellular communications have attracted research attention [13]. A previous study showed that although SPEs isolated from different individuals were similar regarding size, shape, and exosomal markers, their cargos were markedly different [13]. Indeed, SPEs were considered an alternative pathway for intercellular communication by transferring their molecular cargo (DNAs, RNAs, lipids and proteins) to sperm [14]. Guo et al. has reported that the cargo of extracellular ATPs in SPEs regulate sperm motility and mitochondrial metabolism by infiltrating into the sperm membrane [15]. Du et al. demonstrated that inhibition of premature capacitation is likely related to the SPEs transfer proteins including AWN and PSP-1 into sperm [16]. Other studies provided strong evidence that glycogen synthase kinase 3 (GSK3) inhibits sperm motility and proteins of Wnt signaling via exosome mediate the sperm maturation and motility acquisition [17, 18]. Several factors such as temperature, pH, and zinc ion concentrations have been confirmed to affect SPEs transport [19–21]; however, SPEs shuttle mechanism and fusion with the sperm membrane are still unclear.

High-throughput proteomics are crucial for in-depth characterization of SPEs composition. Proteomic studies may help identify potential SPEs biomarkers to diagnose male reproductive disorders [22]. The SPEs proteome was first described to contain 139 proteins, according to liquid chromatography tandem mass spectrometry (LC-MS/MS) in human [23]. The structure of SPEs and proteomic datasets were elucidated, providing important practical information for determining candidate biomarker proteins and understanding the respective biologic functions [24]. A recent proteomic analysis reported 1474 proteins in human SPEs, and gene ontology (GO) analysis showed that SPEs proteins were mostly linked to exosomes, cytoplasm, and cytosol, which are involved in processes such as energy pathways, cell growth, and transport [25].

Buffalo (*Bubalus bubalis*) has adapted to tropical climate conditions but typically show low reproductive efficiency [26]. Male infertility is the major issue impeding buffalo reproduction, and the poor quality of buffalo semen is manifested in low sperm motility, viability and fertility, which may be due to a lesser semen protein concentration [27, 28]. Previously, we identified the proteomes of buffalo spermatozoa and seminal plasma [29]; however, the mechanisms of SPEs interaction with spermatozoa remain poorly understood. To investigate the relationship between buffalo spermatozoa, SP, and SPEs, and to identify key factors related to sperm motility of SPEs in proteomic composition, LC-MS/MS was used on SPEs in buffalo semen with high and low sperm motility. Our results help understand protein differences of spermatozoa, SP, and SPEs, and will be of importance for clarifying the causes of low sperm motility in buffalo and the roles of SPEs in the regulation of sperm maturation and motility, which can be applied to guide the breeding of buffalo fertilization.

## Results

### Isolation and characterization of SPEs

**Fig. 1:**
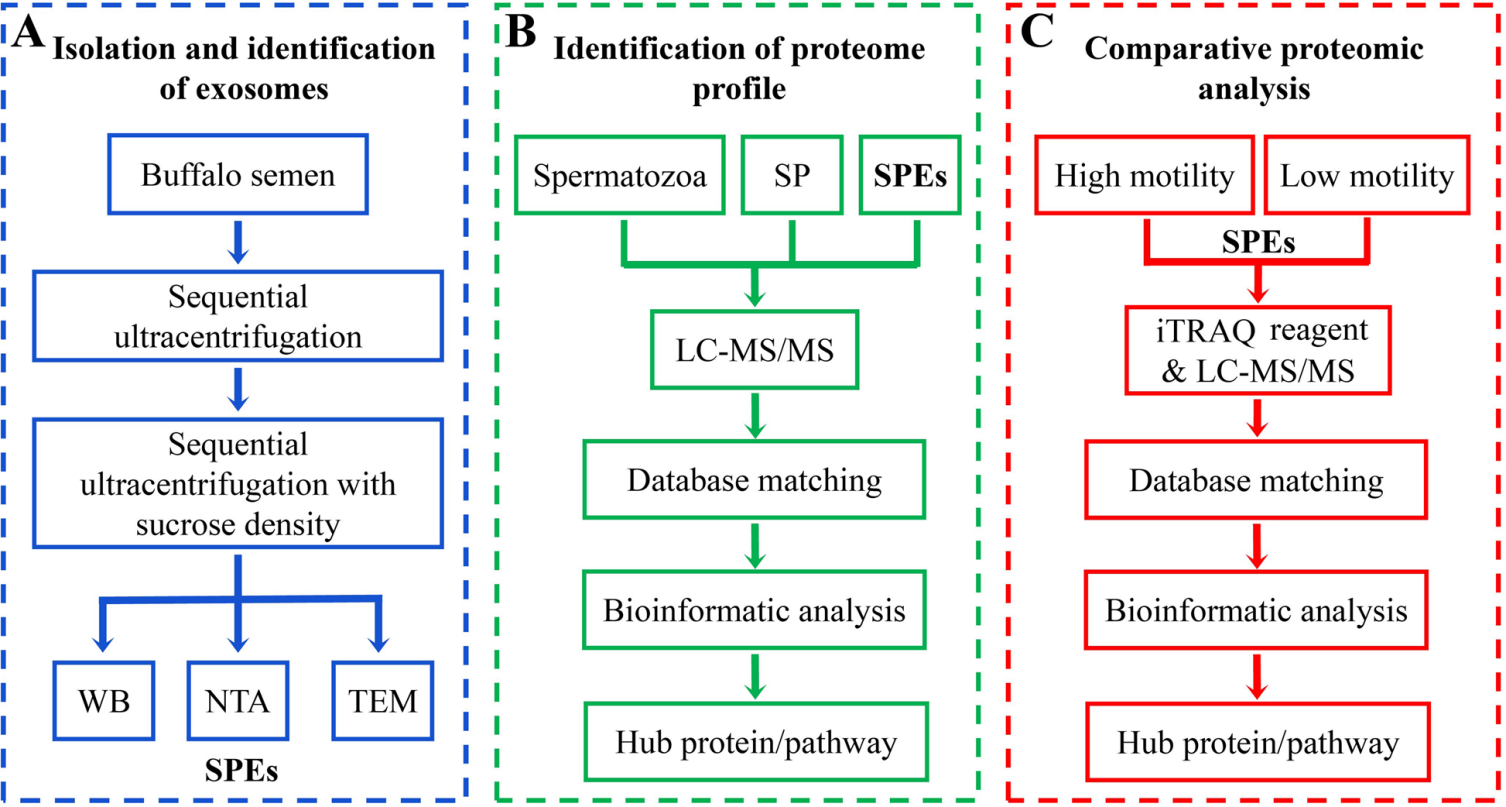
Outline of the preliminary study on the proteome of buffalo seminal plasma exosomes (SPEs). **A** Procedure of exosome isolation. **B** Identification workflow of proteome profile in sperm, seminal plasma, and SPEs samples. **C** Quantification workflow of proteome analysis in H- and L-motility SPEs samples

**Fig. 2:**
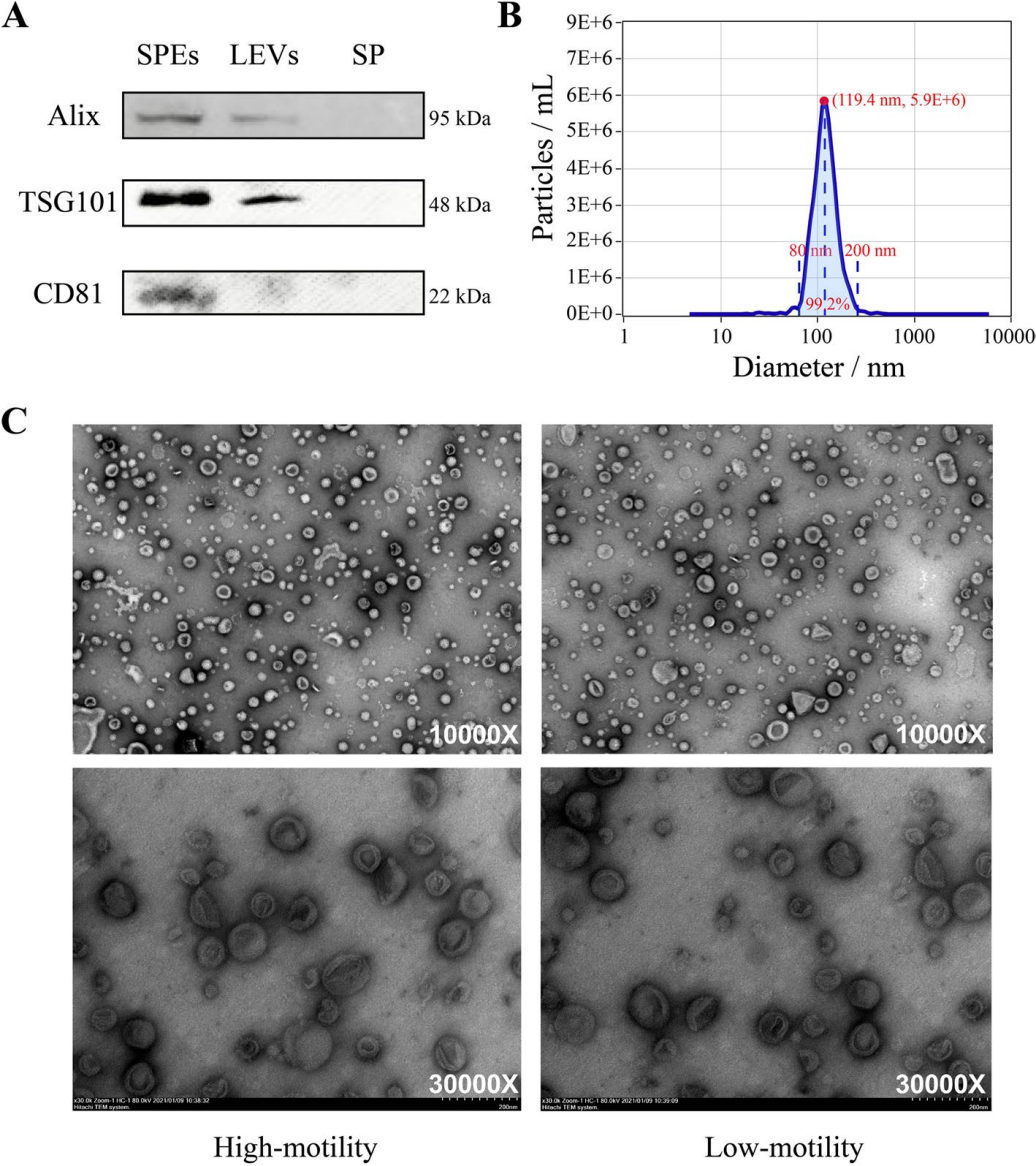
Characterization of seminal plasma exosomes (SPEs) by western blot, nanoparticle tracing analysis (NTA) and transmission electron microscopy (TEM). **A** Protein expression analysis of Alix, TSG101 and CD81 in seminal plasma exosomes (SPEs), large extracellular vesicles (LEVs) and seminal plasms (SP) samples by western blot. The full size image of western blots can be obtained from Additional file 1: Fig. S1. **B** Evaluation of size distribution of SPEs determined by NTA analysis. The red dot indicated particles with size of 119.4 nm have the highest density (5.9E+ 6 particles/mL). The main peak of particles size within 20 ~ 200 nm (blue shaded areas) accounted for 99.2% of all particles. **C** Morphology of isolated SPEs by TEM. The magnification is 10,000× (upper) and 30,000× (lower), respectively

Exosomes were isolated and identified according to the workflow as shown in Fig. 1A. Exosomal marker proteins including Alix, TSG101, and CD81 were validated using western blotting of SPEs, with large EVs (LEVs) and SP as control samples (Fig. 2A and Additional file1: Fig. S1). Three markers were detected in SPEs and LEVs. CD81, TSG101, and Alix proteins produced bands of 22, 48, and 95 kDa, respectively, in purified SPEs samples, whereas no protein expression was observed in SP. Lower expression levels of Alix, CD81and TSG101 were observed in LEVs compared to SPEs. To determine size and concentration of SPEs, Nanoparticle tracing analysis (NTA) analyses were conducted, showing that the SPEs particle diameter ranged from 80 to 200 nm, with the highest peak intensity (5.9 × 10^6^ particles/mL) at 119.4 nm diameter (Fig. 2B). This main peak accounted for 99.2% of all particles. Particle size did not differ significant between the H and L groups.

SPEs morphology was investigated using transmission electron microscopy (TEM) (Fig. 2C), showing their intact cup-shaped structure and membrane integrity. In line with the NTA results, TEM showed that SPEs diameter ranged from 50 to 150 nm. No obvious differences between H- and L-motility group samples were observed. Overall, these results demonstrated that the SPEs were successful isolated and purified from buffalo semen using ultracentrifugation, which had typical characteristics of exosomes.

### Comparisons of proteome profiles

**Fig. 3:**
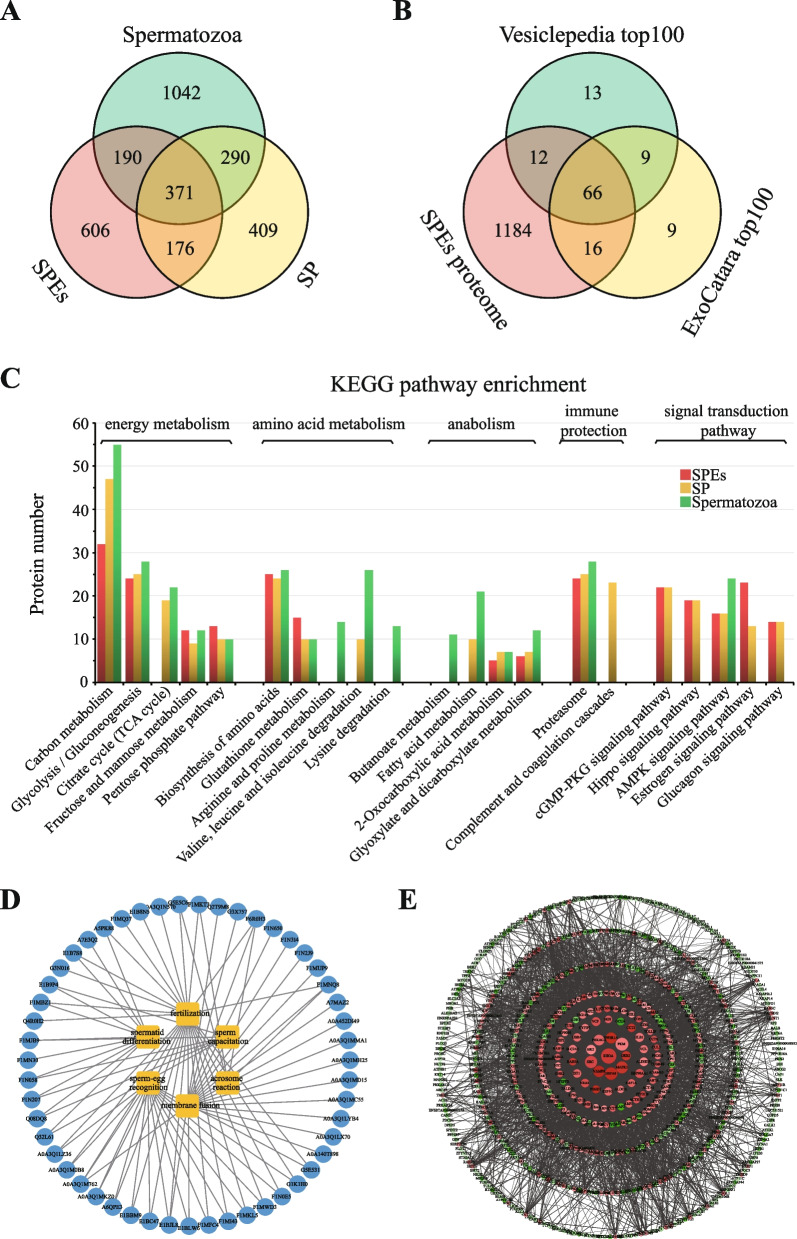
Comprehensive proteome profile analysis of sperm, seminal plasma (SP), and seminal plasma exosomes (SPEs). **A** Comparison of protein expression profiles of sperm, SP, and SPEs in buffalo. **B** Overlapping of exosome proteins compared with extracellular vesicle database Vesiclepedia Topl00 and exosomes database ExoCatar Topl00. **C** Pathway comparison of proteins identified from sperm, SP, and SPEs. **D** SPEs proteins associated with sperm capacitation, spermatid differentiation, membrane fusion, acrosome reaction, sperm-egg recognition, and fertilization. **E** Protein-protein interaction (PPI) network of SPEs. The map size of the node indicates the value of node betweenness, larger nodes are more important in PPI network stability. The shade of the color indicates the degree of the node, red indicates higher than average, green indicates below average. The darker the color, the more proteins it interacts with. The higher gray value of edge means higher score of protein-protein interaction. The network details can be obtained from the original image (Additional file 1: Fig. S3)

To analyze protein expression between spermatozoa, SP, and SPEs, proteome profiles were identified by LC-MS/MS and analyzed bioinformatic methods (Fig. 1B). In total, 1879 proteins were successfully identified in spermatozoa (Additional file 2: Table S1), 1247 proteins in SP (Additional file 2: Table S2), and 1343 proteins in SPEs (Additional file 2: Table S3). A Venn diagram was produced to indicate the 561 proteins shared between spermatozoa and SPEs, 547 proteins shared between SP and SPEs, and 661 proteins shared between spermatozoa and SP. Among all identified proteins, 371 proteins (accounting for 27.6% of the SPEs proteome) were expressed in spermatozoa, SP, and SPEs samples. A total of 606 proteins were unique to SPEs (Fig. 3A). Additionally, proteins identified in SPEs were matched with public exosome databases, and 82 and 79% of the proteins overlapped with the top 100 proteins in the ExoCarta [30–32] and Vesiclepedia [33] databases (Fig. 3B), indicating that the proteome results of SPEs were reliable. Most proteins were commonly expressed in exosomes of different origins. The proteomic data of SPEs (PXD033442) can by fully accessed from the ProteomeXchange consortium via the PRIDE partner repository [34]. To our knowledge, this is the first report on the buffalo seminal exosome proteome.

We compared the annotated GO and Kyoto Encyclopedia of Genes and Genomes (KEGG) pathways of proteins in spermatozoa, SP, and SPEs, showing that proteins mainly participated in GO terms including small GTPase mediated signal transduction, protein transport, intracellular protein transport, calcium ion binding and GTPase activity, and pathways such as amino acid metabolism, immune protection, and signal transduction (Additional file 1: Fig. S2). KEGG enrichment showed significant difference in three samples. SPEs appears to play a central role in immune protection by a pathway involving proteasomes. A series of signaling transduction pathways (cGMP-PKG signaling, Hippo signaling, AMPK signaling, Estrogen signaling) were significantly enriched, suggesting that SPEs may be associated with more complex regulation pathways responsible for signaling transduction involve in sperm development (Fig. 3C). Particularly, the AMPK signaling pathway, associated with maintaining the structure of plasma membrane and the integrity of acrosome membrane, showed that SPEs were closely related to the regulation of spermatogenesis and sperm motility. Additionally, several proteins associated with reproduction were characterized, including 6 proteins annotated to sperm capacitation, 11 proteins annotated to spermatid differentiation, 28 proteins annotated to fertilization, 14 proteins annotated to sperm-egg recognition, 20 proteins annotated to membrane fusion, and 4 proteins annotated to acrosome reaction (Fig. 3D). Protein-protein interaction (PPI) networks were constructed for hub protein identification. The SPEs PPI network comprised 630 nodes and 3846 edges. The top 10 crucial proteins for maintaining the stability of the PPI network are indicated in Fig. 3E and Additional file 1: Fig. S3. These proteins included RHOA, HSPA8, PKM, MAPK1, VAMP8, PPP2R1A, SRC, PRKACA, GRB2, and HK2.

### Analysis of differential expression proteins (DEPs) in SPEs

**Fig. 4:**
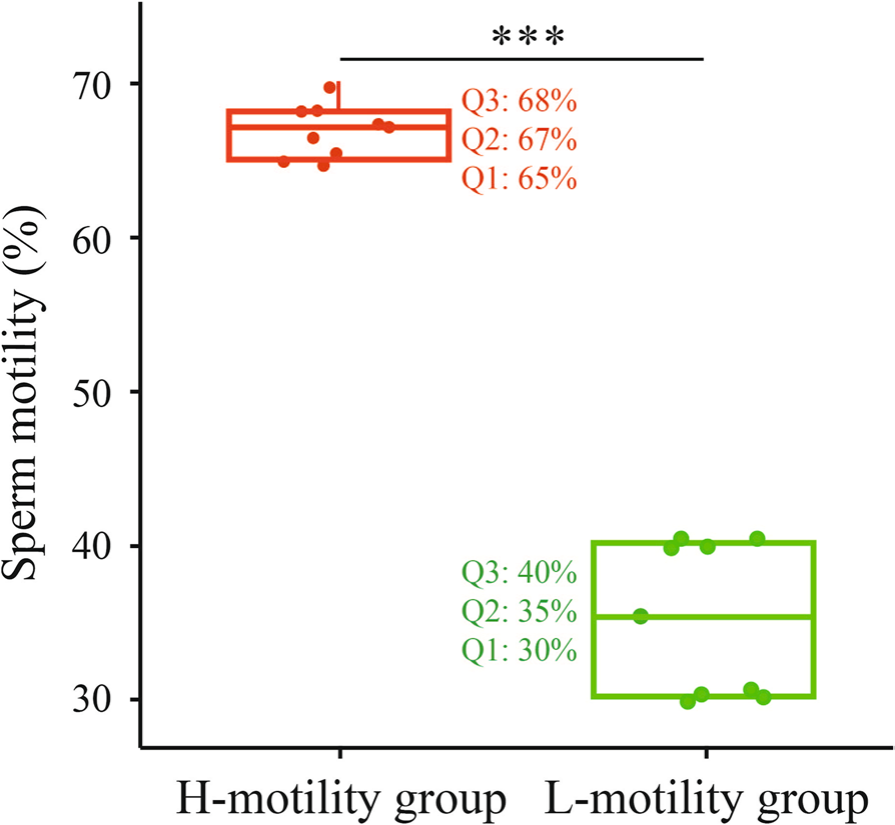
Comparison of sperm motility between H-motility and L-motility SPEs in buffalo. The different color boxplot represents the different groups. The middle line represents the median (Q2) value, the top line represents the upper quartile (Q3) value and the bottom line represents the lower quartile (Q1) value of box. *** indicates significant difference (*P* value < 0.001)

**Fig. 5:**
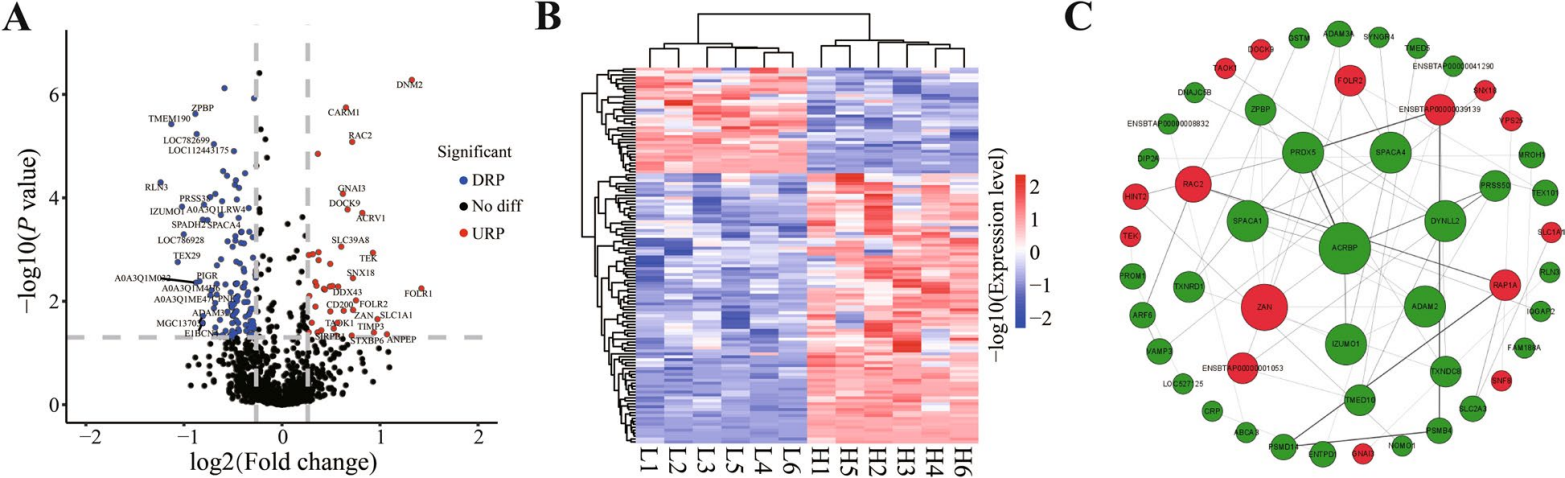
Differential proteomic analysis between H-motility and L-motility seminal plasma exosomes (SPEs) in buffalo. **A** Volcano plot for differentially expressed proteins (DEPs) screening. The top 20 up-regulated proteins (URPs) (red dots) and down-regulated proteins (DRPs) (blue dots) labeled with protein names or Uniprot IDs. The thresholds of fold change were set as 1.2 and 1/1.2. *P* value < 0.05 was defined as statistically significant. **B** Heatmap of DEP expression levels of each sample. **C** Protein-protein interaction network of DEPs. Red nodes indicate URPs, and green nodes indicate DRPs. Node size indicates the value of node betweenness; larger nodes are more important in PPI network stability. The higher gray value of the edge indicates a higher score of protein-protein interaction

A comparative proteomic analysis based on iTRAQ-label coupled with LC-MS/MS were carried out between H-motility group and L-motility group sperm (Fig. 1C and Fig. 4). To clarify the proteins contributing to sperm motility, comparative proteomic analysis of SPEs was carried out to identify the DEPs. Complete protein lists of DEPs with accession numbers, protein names, and sequence coverage are presented in Additional file 2: Table S4. Compared to H-motility group SPEs, a total of 160 DEPs were found in L-motility group SPEs (fold change ≥1.2), including 119 down-regulated DEPs and 41 up-regulated DEPs (Fig. 5A), which showed a general downward trend of protein expression level. A heatmap of DEP expression levels suggested good accuracy and reproducibility among biological replicates (Fig. 5B). In addition, the PPI network of DEPs indicated that several proteins such as ACRBP, SPACA1, PRDX5, SPACA4, DYNLL2, ZAN, IZUMO1, and ADAM2 in SPEs may play an important role in modulating sperm motility (Fig. 5C).

### Functional analysis of DEPs in SPEs

**Fig. 6:**
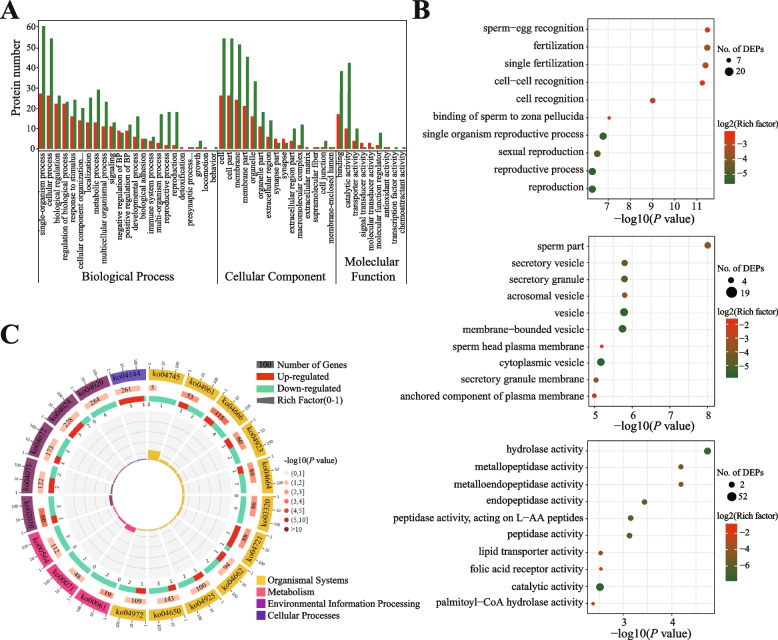
Functional analysis of differential expression proteins (DEPs) between H-motility and L-motility seminal plasma exosomes (SPEs) in buffalo. **A** Gene Ontology (GO) classification of up-regulated and down-regulated DEPs in L-motility group SPEs in comparison with H-motility group SPEs. **B** GO enrichment map (top 10) of significant vesicle of DEPs. **C** Enrichment circle diagram of Kyoto Encyclopedia of Genes and Genomes (KEGG) pathway of DEPs

To investigate the main functions of DEPs in SPEs, enriched Gene Ontology (GO) terms were classified (Additional file 2: Table S5, S6, and S7). GO classification indicated that down-regulated DEPs were mainly located in the membrane, extracellular region, and macromolecular complexes of cellular components. Regarding biological processes, numerous down-regulated DEPs were associated with metabolic, reproductive, and developmental processes (Fig. 6A). GO enrichment analysis showed that DEPs were located in sperm and vesicles, with activities of hydrolase and metalloproteinase, and were involved in sperm-egg recognition, fertilization, single fertilization, sperm-zona pellucida binding, monobiotic reproduction, cell recognition, and other processes (Fig. 6B). Furthermore, KEGG enrichment results showed that DEPs were mainly involved in the PPRP signaling, calcium signaling, and cAMP signaling pathways, among others (Fig. 6C), and the expression levels of 10 proteins such as IZUMO1 were significantly down-regulated in L-motility group SPEs (Table 1 and Additional file 2: Table S8).

**Table 1:** KEGG pathways with significant enrichment of DEPs

KEGG ID	Pathway	*P* value	Counts	Genes
ko04666	Fc gamma R-mediated phagocytosis	0.000	6	DNM2,RAC2,ARF6,N/A,N/A,PLA2G6
ko02010	ABC transporters	0.001	4	LOC524391,LOC786628,ABCA3,ABCA5
ko03320	PPAR signaling pathway	0.004	4	PDPK1,N/A,ACSBG2,AQP7
ko04664	Fc epsilon RI signaling pathway	0.004	4	PDPK1,RAC2,N/A,N/A
ko04721	Synaptic vesicle cycle	0.004	4	SLC1A1,DNM2,ATP6V0D1,SYT1
ko04961	Endocrine and other factor-regulated calcium reabsorption	0.007	3	ATP2B1,DNM2,ATP2B4
ko00061	Fatty acid biosynthesis	0.008	2	N/A,ACSBG2
ko04923	Regulation of lipolysis in adipocyte	0.009	3	PTGS1,GNAI3,AQP7
ko04144	Endocytosis	0.010	6	SNF8,FOLR1,FOLR2,DNM2,VPS25,ARF6
ko04071	Sphingolipid signaling pathway	0.012	4	GNAI3,PDPK1,RAC2,BID
ko04020	Calcium signaling pathway	0.012	6	ATP2B1,ATP2B4,ENO,ENO,PLCD4,PDE1C
ko04650	Natural killer cell mediated cytotoxicity	0.018	4	RAC2,N/A,N/A,BID
ko04024	cAMP signaling pathway	0.021	5	ATP2B1,GNAI3,ATP2B4,RAC2,RAP1A
ko04662	B cell receptor signaling pathway	0.024	3	RAC2, N/A,N/A
ko04745	Phototransduction - fly	0.026	1	PPEF1
ko04925	Aldosterone synthesis and secretion	0.026	3	ATP2B1,ATP2B4,SCARB1
ko04072	Phospholipase D signaling pathway	0.028	4	DNM2,ARF6
ko04972	Pancreatic secretion	0.029	3	ATP2B1,ATP2B4,RAP1A
ko00071	Fatty acid degradation	0.032	2	N/A,ACSBG2
ko00564	Glycerophospholipid metabolism	0.034	3	GPD1L,PLA2G

### Parallel reaction monitor (PRM) validation of DEPs

**Fig. 7:**
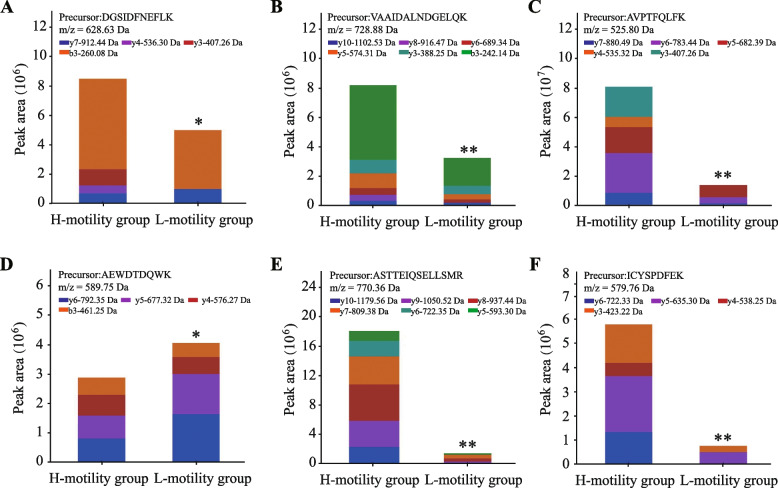
Parallel reaction monitoring (PRM) validation of differentially expressed proteins (DEPs). **A** PPEF1 protein. **B** ST13 protein. **C** TXNDC8 protein. **D** LRRC37 protein. **E** SPACA1 protein. **F** GSTM2 protein. * indicates significant difference (*P* value < 0.05); ** indicates significant difference (*P* value < 0.01)

Targeted PRM analysis was conducted to provide relative peptide quantification for validation. Six proteins were detected and significant differential expressions were confirmed, namely, PPEF1, ST13, TXNDC8, LRRC37AB, SPACA1 and GSTM2 (peptide information and peak areas are shown in Additional file 2: Table S9). As shown in Fig. 7, five of these proteins (i.e., PPEF1, ST13, TXNDC8, SPACA1, and GSTM2) (Fig. 7A-C, E, F) were down-regulated and LRRC37 (Fig. 7D) was up-regulated in SPEs of L-motility group. The result exhibited the same trend in the proteomic dataset.

## Discussion

Exosomes facilitate intercellular communication by entering target cells via surface ligands. The cellular effectors enclosed inside or on the surface membrane such as mRNA, miRNA and origin proteins, can be transferred between exosomes and recipient cells [35]. Considering that spermatozoa are transcriptionally quiescent cells, the acquisition of new products during final maturation in seminal plasma may rely on transport by SPEs [36]. Exosomes also play important roles in inflammatory and immune protection during sperm migration in the female tract [37]. It is thus important to establish a reliable method to harvest SPEs and to further assess interaction between SPEs and spermatozoa. Ultracentrifugation and sucrose gradient centrifugation were confirmed in this study as efficient methods to isolate and purify SPEs. SPEs showed cup-shaped morphology, however, TEM images showed no difference between H- and L-motility group SPEs. The cup shape was an artefact form due to the drying process, as cryo-TEM confirmed a perfectly spherical structure of exosomes in aqueous solution [38].

Proteomics are a promising strategy for assessing the protein composition and differences in SPEs. Numerous high-throughput proteomic studies based on LC-MS/MS assessed the protein composition of spermatozoa and SP, and potential diagnostic biomarkers of male infertility were identified in multiple mammalian species such as humans and mice. In our previous study, the proteome landscape of spermatozoa and SP in buffalo were identified through comprehensive proteomic analyses [29]. We characterized several highly abundant proteins and focused on functional analysis of overlapping and unique proteins; however, the protein correlation between spermatozoa, SP, and SPEs remain unclear. Additionally, the crucial proteins associated with sperm maturation, motility, and male fertilization capacity in buffalo remain to be examined in detail. In the current study, the proteome profiles were analyzed regarding differences between spermatozoa, SP, and SPEs. Furthermore, proteomic differences between SPEs of H-motility and L-motility sperm were compared.

The proteomic results showed that 561 SPEs proteins (41.8%) were also found in spermatozoa, and 547 SPEs proteins (40.7%) were found in SP. Nearly one-third of SPEs proteins (371, 27.6%) were present in spermatozoa and SP. These proteins may play an important role in the microenvironmental balance regarding sperm maturation. GO annotation results of cellular components also showed that extracellular exosome proteins were most abundant in spermatozoa, SP and SPEs. Consistent with the biological process annotation results, proteins participated in oxidation-reduction process were mostly attributed to biological process in spermatozoa and SP. In SPEs, proteins participating in small GTPase-mediated signal transduction were most abundant, which is closely related to sperm capacitation and fertilization [39]. GTPases affect components of the plasma membrane, the nucleus, and the endomembrane system, which are involved in intracellular actin/tubulin-dependent vesicle movement, membrane fusion, and cell growth during mitosis. Abnormal expression of GTPases disrupts intracellular systems. In spermatozoa, GTPases are vital for cytoskeletal organization [40]. Further studies are needed to elucidate how SPEs mediate sperm motility through small GTPase signal transduction (Fig. S2A). Ca^2+^ ions, a main type of second messenger molecules mediating sperm movement, are strictly regulated in the intracellular and extracellular space during the maturation of sperm in epididymis [41]. Moreover, 64 and 60 proteins were assigned to Ca^2+^-binding capacity in spermatozoa and SPEs, respectively (Fig. S2B), of which 33 proteins were shared.

Among the identified SPEs proteins, functional proteins related to reproduction were analyzed. We found 21 proteins involved in spermatogenesis. ROPN1L, a protein participating in maintaining the integrity of fiber sheath and sperm motility, plays an important role in PKA-dependent signaling, which is required for sperm capacitation. Fiedler et al. [42] confirmed that mutations of ROPN1 and ROPN1L diminish fiber integrity, thus reducing sperm motility through the PKA signaling pathway and resulting in male sterility. Sixteen SPEs proteins were attributed to sperm-egg recognition, including the highly conserved protein SPA17, which plays an important role in regulating sperm maturation, capacitation, acrosome reaction, and interaction with the oocyte zona pellucida [43]. Additionally, a total of 25 RAB family members were identified in SPEs. The RAB protein family is the largest branch of the small GTPase superenzyme family. As a molecular switch regulating intracellular transport in eukaryotic cells, RAB proteins are crucial regulators of signal transduction, cell growth, and differentiation [39]. RAB proteins may be related to acrosome formation during spermatogenesis and acrosome reaction during fertilization [44]. Moreover, synthetic peptides of RAB3 can lead to acrosome rupture [45]. Particularly, RAB27B is known to facilitate interactions of myosin-Va with the vesicle, and immunoreactive sites can be observed in the medullary region of the Golgi apparatus and in the adjacent acrosome [44]. Considerable amounts of RAB proteins were differentially expressed in SPEs, suggesting that exosomes may mediate acrosome formation and the acrosomal reaction. Other proteins associated with sperm motility including MIF, ATP2B4, GSK3, ELSPBP1, GLIPR1L1, etc. were also observed in SPEs. MIF is transported to spermatozoa through exosomes and binds to peripheral dense fibers to eliminate Zn^2+^ bound to sperm flagella, thus affecting sperm motility [46]. ATP2B4, an ubiquitous Ca^2+^ pump, can regulate the activation of the Ca^2+^ signaling pathway to maintain a steady state of Ca^2+^ in sperm. The Ca^2+^ balance directly affects sperm motility. Furthermore, sperm motility can also be regulated by GSK3, which participates in the Toll-like receptor and Wnt signaling pathways [47]. In the presence of Zn^2+^, ELSPBP1/BLVRA complexes bind to dead sperm to prevent adverse effects of reactive oxygen species produced by dead sperm cells [48]. A member of the CAP superfamily, GLIPR1L1, is located in the sperm head through epididymal corpuscles and participates in the recognition process of sperm and egg, together with other family members [49].

KEGG pathway analysis showed that the identified proteins were enriched regarding the AMPK signaling pathway in sperm, SP, and SPEs. The AMPK signaling pathway was first confirmed to be related to spermatogenesis and regulation of sperm motility in 2012 [50], and it is involved in the main physiological functions of mature sperm, especially regarding the maintenance of sperm motility, sperm membrane integrity, and mitochondrial membrane potential. Energy balance is necessary for the production and quality of sperm, and it is linked to the hypothalamic-pituitary-gonadal axis through the core protein AMP-activated protein kinase in the AMPK signaling pathway [51]. The effect of AMPK on mitochondrial membrane potential depends on the extracellular stimulation of sperm cells. AMPK transmits this stimulation signal to affect sperm energy metabolism and maintain the membrane potential of sperm mitochondria, so as to promote normal movement of sperm. Sperm fertilization capacity depends on the sperm plasma membrane and its lipid fraction. AMPK plays an important role in plasma membrane fluidity and in maintaining the lipid organization of sperm in pigs [52] and goats [53].

Differential proteomics of SPEs showed 160 significantly differentially expressed proteins, including 41 up-regulated and 119 down-regulated DEPs in L-motility group SPEs, compared with those of the H group. The overall decreasing trend of protein expression in L-motility group samples was consistent with the results in buffalo seminal plasma, human seminal plasma and spermatozoa [54]. GO cellular component enrichment results showed that 11 DEPs (10 down-regulated and 1 up-regulated) were annotated to sperm parts in low-motility SPEs, including IZUMO1, ACRBP, SPACA1, ZPBP, ACRV1 etc. Of these, IZUMO1 is an essential protein during sperm-egg fusion. IZUMO1-deficient spermatozoa can cross the zona pellucida normally, but are unable to fuse with a normal oocyte [55]. During spermatogenesis, ACRBP participates in the formation of acrosome particles by maintaining the inactive state of acrosin before acrosome exocytosis, and knockout of the *Acrbp* gene leads to severely decreased fertility in mice [56]. The SPACA1 protein is located in the equatorial segment of sperm and plays a role in the sperm-oocyte fusion process. Knockout of the *Spaca1* gene leads to a deformed sperm head, resulting in infertility in mice [57]. ZPBP, an important acrosome matrix component of sperm, functions in the process of sperm crossing the zona pellucida. Mice lacking *Zpbp1* gene show abnormal sperm morphology, broken acrosomes, and sperm disability of progressive movement [58]. The acrosome matrix protein ZACRV1 (SP-10) is conserved in mammals and exerts a crucial role in the adhesion or penetration between sperm and oocytes. Therefore, we speculate that these proteins in SPEs can be transferred to sperm, which can affect sperm motility and contribute to the process of fertilization. GO molecular function enrichment analysis showed that 10 DEPs (9 down-regulated and 1 up-regulated) were assigned to metallopeptidase activity in L-motility group SPEs. Three metalloprotease-disintegrin proteins (ADAM2, ADAM3, and ADAM32) were among the down-regulated DEPs. ADAM2-ADAM3 complexes are important for various function of sperm in mice, including sperm-sperm aggregation, sperm oocyte interaction, and sperm entry of the fallopian tube from the uterus [59]. However, male *Adam32* knockout mice show normal fertility, testicular integrity, and sperm characteristics [60]. Therefore, we speculate that SPEs can affect the normal function of sperm by transferring the ADAM2-ADAM3 complex. GO biological process enrichment showed that 10 DEPs (8 down-regulated DEPs and 2 up-regulated DEPs) participated in the process of sperm-oocyte recognition in L-motility group SPEs. Among them, testicular expression protein 101 (TEX101), a cancer/testicular antigen family member, was initially identified in mouse testes and was subsequently found to be expressed in human and bovine germ cells [61, 62]. During fertilization in mice, protein TEX101 acts on cumulus cells after removal from sperm, causing cumulus cells to elicit Ca-dependent progesterone release, thus initiating the acrosome reaction [63]. *Tex101* gene knockout mice show normal sperm morphology and concentrations in ejaculate, but exhibit the loss of adhesion ability, resulting in a failure of sperm-oocyte fusion. In addition, *Tex101* gene knockout markedly reduces or prevent the expression of ADAM3 [64, 65] in spermatozoa. The expression level of TEX101 protein is significantly decreased in the seminal plasma of azoospermia patients, compared with normal males, and the concentration of TEX101 in seminal plasma is related to the number of testicular germ cells [61]. Therefore, we speculate that SPEs affect spermatogenesis and development by participating in the sperm-anchoring and sperm-shedding process of TEX101. The expression level of TEX101 in SPEs may thus be potential biomarker of male sterility.

Based on the results of KEGG pathway enrichment, we found that 4 proteins belonging to the PPAR signaling pathway and 6 proteins assigned to the Ca signaling pathway were differentially expressed. PPARs are ligand-dependent transcriptional regulators that affect a wide range of intracellular metabolism processes, especially lipid metabolism. PPARγ, a subtype of PPARs, is closely related to the reproductive physiological function of males, and it plays a crucial role in spermatogenesis by participating in lipid metabolism in Sertoli cells [66], participating in fertilization by providing energy [67], and participating in sperm capacitation through glucose metabolism or other metabolic processes [68]. Aquaporin-7 (AQP7) is an important member involved in the PPAR signaling pathway, which is expressed in kidney, heart, testicular, and adipose tissue, and it is involved in glycerol transport and cytoplasmic concentration during spermatogenesis in mammals [69]. AQP7 is expressed in human sperm cells and also in sperm tail. Moreover, the motility of sperm in AQP7-expressing males is markedly higher than that in the negative expression population [70], and AQP7 expression is significantly positively correlated with the proportion of normal sperm cells [71]. Consistent with these results, we found that AQP7 expression of was significantly lower in low-motility SPEs, suggesting that AQP7 in SPEs may have an important effect on sperm motility. However, the specific mechanism needs to be further studied. Sperm motility is closely related to Ca^2+^ concentrations. Plasma membrane Ca ATPase 4 (ATP2B4) belongs to the P-type ATPase family, and it is an important Ca^2+^ pump in the Ca signaling pathway. Sperm motility is severely impaired in ATP2B4 knockout mice and mice treated with ATP2B4 inhibitor, indicating that ATP2B4 regulates sperm motility [72]. Epididymal can transfer ATP2B4 to sperm through secreted exosomes during the maturation of sperm in the epididymis, resulting in higher ATP2B4 concentrations in the sperm tail than in the head [73]. Our result showed that ATP2B4 expression in low-motility SPEs was significantly decreased, suggesting that ATP2B4 in SPEs may affect sperm motility though specific mechanisms which, however, remain unclear. We suggest that SPEs may transfer ATP2B4 to sperm by membrane fusion, thus affecting sperm motility.

In conclusion, we established the proteome profile and the relationship between spermatozoa, SP and SPEs in buffalo. Moreover, the differential proteomics of SPEs between H- and L-motility groups were identified. The annotated biological functions and metabolic pathways were further analyzed, and a number of functional proteins related to spermatogenesis, maturation and fertilization were obtained by comprehensive bioinformatic analysis. This study extends our understanding of seminal plasma exosomes and provides clues for determining the regulatory mechanisms of seminal plasma exosomes in buffalo sperm motility.

## Materials and methods

### Sample collection

Buffalo semen samples were collected through artificial ejaculation at the Livestock Breed Improvement Station of Guangxi province, China. The semen samples were divided into two groups (*n* = 9 animals per group): group I (motility: 66.78 ± 1.72% (mean ± standard error), a median of 67%; defined as H-motility group) and group II (motility: 36.11 ± 6.97% (mean ± standard error), a median of 35%; defined as L-motility group) as shown in Fig. 3. Semen quality was analyzed using Computer Assisted Sperm Analysis (CASA) software [74]. The animal experiments were approved and monitored by the Animal Experiments Ethical Review Committee of Guangxi University (approval code: GXU2020–037), The study was performed in accordance with the ARRIVE Guidelines for reporting animal research [75]. We declared that all methods were carried out in accordance with animal ethics guidelines and regulations.

### Isolation and purification of SPEs

Buffalo SPEs were isolated by ultracentrifugation, purified by sucrose density gradient centrifugation as previously described [76], with minor modifications. Briefly, liquefied semen samples were subjected to sequential centrifugation steps at 800×g for 10 min to remove sperm cells, at 4000×g for 30 min to remove cell fragments, and at 12,000×g for 45 min to eliminate large vesicles. Subsequently, the supernatant was filtered through 0.22-μm filters after dilution with phosphate-buffered saline (PBS). Then, the medium containing SPEs was centrifugated at 100,000×g for 90 min using an ultracentrifuge (Optima XL-100 k; Beckman, USA). The resulting precipitates were re-suspended using PBS and were underlayered with 30% sucrose cushion solution. After centrifugation at 130,000×g for 90 min, the sucrose cushion was washed with PBS, and centrifugation was performed at 100,000×g for 90 min. Finally, the concentrated SPEs were re-suspended using 100 μL PBS and were frozen at − 80 °C until subsequent analyses.

### NTA

The size and concentration of SPEs were determined using an NTA device (ZetaView, Germany) as described by Gardiner et al. [77]. SPEs were diluted with 1 mL Milli-Q water (Merck Millipore, USA). Each sample was analyzed for 60 s and was measured three times. A power spectral density plot was created with.

estimated particle diameter (nm) indicated on the x-axis and particle concentration (particles/mL) on the y-axis.

### TEM analysis

TEM analysis was performed to assess SPEs morphology as described previously [25] with minor modifications. First, SPEs samples were diluted using PBS, and 10 μL of the solution was transferred to a copper grid for incubation for > 20 min at room temperature (RT). Then, SPEs samples were stained using 1% acetic acid for 3 min in darkness. After drying at RT for 20 min, the samples were examined using a transmission electron microscope instrument (HT-7700, Hitachi, Japan).

### Western blotting

For identification of exosome marker proteins through western blotting, total proteins of SPEs, LEVs and SP were extracted using Urea-EDTA-DTT (UED) lysis buffer (8 M urea, 2 mM ethylenediaminetetraacetic acid (EDTA), 10 mM dithiothreitol (DTT)) with 1% protease inhibitor cocktail. Protein concentration was determined using a BCA protein quantitation kit (Cat No. P0012S, Beyotime, China) according to the manufacturer’s instructions. For each sample, 10 μg protein was loaded on an 12% SDS-PAGE gel, followed by separation for 90 min in the electrophoresis buffer (25 mM Tris-base, 192 mM glycine, 0.1% SDS). Proteins were transferred to 0.22-μm polyvinylidene fluoride (PVDF) membranes (Merck Millipore, Germany) using the transfer buffer (48 mM Tris-base, 39 mM glycine, 20% methanol) at 40 V for 90 min. Then, the membranes were incubated with 5% (w/v) skimmed milk for blocking for > 1 h and were washed three times using Tris buffered saline with 0.1% tween 20 (TBST). After diluting the samples to working concentration, primary antibodies (CD81, sc-166,029, dilution 1:500; Alix, sc-53,538, dilution 1:500; TSG101, sc-7964, dilution 1:500; Santa Cruz, USA) were added to membranes for overnight incubation in 4 °C, respectively. After washing three times using TBST, the membranes were incubated with horse anti-mouse secondary antibody (Cat No. A0258, dilution 1:5000, Beyotime, China) for 2 h. Thereafter, the membranes were washed three times using TBST, incubated with a BCIP/NBT kit (Cat No. C3206, Beyotime, China), and were assayed using Gel Imaging scanner (Amersham Imager 600, GE Healthcare, USA).

### Protein extraction, digestion, labeling, and pre-fractionation

For protein extraction, the protocol was described in our previous publication [78]. UED buffer with 1% protease inhibitor cocktail was added to spermatozoa, SP, and SPEs samples for lysis at 4 °C for 30 min. After sonication for 2 min, the samples were centrifuged at 10,000×g and 4 °C for 30 min. Protein supernatants were collected and were mixed with three volumes of acetone for precipitation. The protein pellets were re-suspended using Urea-TEAB (UT) buffer (8 M urea and 100 mM triethylammonium bicarbonate (TEAB)), and after dilution with Milli-Q water (Merck Millipore), the concentrations of protein samples were measured using a BCA protein quantitation kit (Beyotime, China).

A total of 100 μg protein per sample was used for proteomic analyses. First, protein was reduced with 10 mM DTT for 45 min at 37 °C, alkylated with 25 mM iodoacetamide for 15 min at RT in darkness. Then, a moderate amount of 100 mM TEAB was added to reduce the concentration of urea, and protein digestion was achieved through added 2 μg trypsin for 16 h, and thereafter, 1 μg trypsin was added for an additional digestion step.

After digestion, the resulting peptides were desalted using a Strata X SPEs column (Phenomenex, USA) according to the manufacturer’s instructions. For isotope labeling, the dried peptides were re-dissolved using 500 mM TEAB and were allowed to react with isobaric tags for relative and absolute quantitation (iTRAQ) Reagent-8Plex (Cat No. 4390812, AB Sciex, USA) for 2 h with shocking at RT. Nine samples in each group were divided into three biological replicates (each for 3 individuals) randomly. For each replicate, H-motility group samples were labeled with iTRAQ-113, and L-motility group samples were labeled with iTRAQ-114. Then, the labeled peptides of the two groups were mixed and dried of each replicates. The samples were then dissolved in solvent A (2% ACN, pH 10) and were injected into the liquid chromatography separation systems (HPLC) system (Waters e2695; Waters, USA) for separating fractions using a high-pH reverse-phase C18 column (130 Å, 3.5 μm, 4.6 × 250 mm; XBridge Peptide BEH C18, Waters, USA) [29]. Binding peptide was eluted using a gradient as described by Lin et al. [78]. For proteomic analysis of spermatozoa, SP, and SPEs, the peptides were divided into six fractions and were desalted using Ziptip C18 Tips (ZTC18S096, Merck Millipore).

Samples of proteome identification and proteomic comparison were analyzed using a LC-MS/MS system comprising an Easy-nanoLC coupled with an LTQ-Orbitrap Elite (Thermo Fisher Scientific, USA) as described in our previous study [79] with minor modifications. Briefly, each peptide fraction was loaded on a reversed-phase trap column (Acclaim PepMap®100 C18, 3 μm, 100 Å, 75 μm × 2 cm) and was then transferred to a reversed-phase analytical column (Acclaim PepMap® RSLC C18, 2 μm, 100 Å, 50 μm × 15 cm). A 68-min elution gradient from 2 to 98% solvent B at 300 nL/min was used for mass spectrometric analysis. The acquired precursor ions ranging from 300 to 1800 m/z were detected using an orbitrap analyzer at a resolution of 70,000. The top 20 abundant precursor ions were fragmented in a selection window of 2 m/z using higher-energy collisional dissociation (HCD) mode at 39% normalized collision energy. Fragment ions were also analyzed using an orbitrap at a resolution of 17,500 and with a scan range of 100+ m/z. The dynamic exclusion duration was set at 30 s, and the ion 445.12003 (m/z) was used for lock mass.

### Data processing and bioinformatic analyses

The peptide mass spectrum was searched against the Uniprot database of *Bos taurus* (UP000009136) using SEQUEST software integrated in the Proteome Discoverer platform (version 2.1; Thermo Fisher Scientific, USA). Trypsin/P was selected as the working enzyme. The max missed cleavage was set to two. Carbamidomethyl (C) was set as fixed modification, and iTRAQ 8plex (N-term, K, Y) and oxidation (M) were set as variable modifications. The peptide mass error was limited to 10 ppm, and the product ion mass error was set to include 0.02 Da. The peptide false distribution rate is reported at *p* < 0.05.

For protein functional analysis, Database for Annotation, Visualization and Integrated Discovery (DAVID) software version 6.8 was used for GO annotation [80]. The online tool Kyoto Encyclopedia of Genes and Genomes (KEGG) [81] was used for pathway annotation. Protein interaction information was obtained from the STRING database, and the interaction network was visualized using Cytoscape software (version 3.4) [82].

### PRM validation

PRM analysis for protein expression validation was conducted using Skyline software (version 21.2) [83]. Pooled samples were separated using an LC-MS/MS system comprising a nanoLC device coupled with an LTQ-Orbitrap Elite and using a 120-min elution gradient for protein library construction. PRM raw files were imported into Skyline software, and the FASTA protein file to identify exosomes was used as background. A total of eight precursors, representing six significantly DEPs, were selected based on the predetermined criteria (i.e., higher peak intensity, no oxidation modification, no missing cleavage site). The targeted precursors were acquired using data-dependent acquirement mass spectrometry mode.

### Statistically analysis

Statistical analyses were performed using the Statistical Package for the Social Sciences software (SPSS) version 19. Statistical analyses of sperm motility, PRM validation were performed using Student’s *t*-tests. A *P* value of 0.05 was regarded as significant. For protein expression analysis, the mean data was used for calculate the fold change. Proteins with fold changes (mean value of triplicates) of > 1.2 or < 1/1.2 with *t*-test *P* values < 0.05 for samples were considered significantly regulated.

## Supplementary Information

**Additional file 1:** **Fig. S1.** The full size image of western blots of Alix, TSG101 and CD81 in seminal plasma exosomes (SPEs), large extracellular vesicles (LEVs), and seminal plasma (SP) samples. **Fig. S2.** Functional classification based on Gene Ontology (GO) annotation of proteome profile in spermatozoa, seminal plasma (SP), and seminal plasma exosomes (SPEs). **Fig. S3.** Original image of protein-protein interaction (PPI) network of seminal plasma exosomes (SPEs).

**Additional file 2:** **Table S1.** Proteome profile of spermatozoa. **Table S2.** Proteome profile of SP. **Table S3.** Proteome profile of SPEs. **Table S4.** DEP list of SPEs. **Table S5.** GO_BP enrichment of DEPs. **Table S6.** GO_MF enrichment of DEPs. **Table S7.** GO_CC enrichment of DEPs. **Table S8.** KEGG enrichment of DEPs. **Table S9.** Peak area of PRM validation.

## Data Availability

The proteomic data of SPEs can be fully accessed from the ProteomeXchange consortium via the PRIDE partner repository (https://www.ebi.ac.uk/pride/archive/projects/PXD033442).

## Abbreviations

SPEs: Seminal plasma exosomes
SP: Seminal plasma
GO: Gene ontology
KEGG: Kyoto Encyclopedia of Genes and Genomes
EVs: Extracellular vesicles
LEVs: Large EVs
LC-MS/MS: Liquid chromatography tandem mass spectrometry
NTA: Nanoparticle tracing analysis
TEM: Transmission electron microscopy
PPI: Protein-protein interaction
DEPs: Differential expression proteins
PRM: Parallel reaction monitor
CASA: Computer Assisted Sperm Analysis
PBS: Phosphate-buffered saline
RT: Room temperature
DTT: Dithiothreitol
EDTA: Ethylenediaminetetraacetic acid
TEAB: Triethylammonium bicarbonate
UED: Urea-EDTA-DTT
PVDF: Polyvinylidene fluoride
TBST: Tris buffered saline with tween 20
iTRAQ: Isobaric tags for relative and absolute quantitation
HPLC: Liquid chromatography separation systems
HCD: Higher-energy collisional dissociation
DAVID: Database for Annotation, Visualization and Integrated Discovery

## References

[1] Thery C, Zitvogel L, Amigorena S (2002). Exosomes: composition, biogenesis and function. Nat Rev Immunol.

[2] Pluchino S, Smith JA (2019). Explicating exosomes: reclassifying the rising stars of intercellular communication. Cell..

[3] Jeppesen DK, Fenix AM, Franklin JL, Higginbotham JN, Zhang Q, Zimmerman LJ, Liebler DC (2019). Reassessment of exosome composition. Cell..

[4] Drabovich AP, Saraon P, Jarvi K, Diamandis EP (2014). Seminal plasma as a diagnostic fluid for male reproductive system disorders. Nat Rev Urol.

[5] Moura AA, Chapman DA, Koc H, Killian GJ (2007). A comprehensive proteomic analysis of the accessory sex gland fluid from mature Holstein bulls. Anim Reprod Sci.

[6] Rath D, Knorr C, Taylor U (2016). Communication requested: boar semen transport through the uterus and possible consequences for insemination. Theriogenology..

[7] Milardi D, Grande G, Vincenzoni F, Messana I, Pontecorvi A, De Marinis L (2012). Proteomic approach in the identification of fertility pattern in seminal plasma of fertile men. Fertil Steril.

[8] De Lazari FL, Sontag ER, Schneider A, Moura AAA, Vasconcelos FR, Nagano CS (2019). Seminal plasma proteins and their relationship with sperm motility and morphology in boars. Andrologia..

[9] Kelly VC, Kuy S, Palmer DJ, Xu Z, Davis SR, Cooper GJ (2006). Characterization of bovine seminal plasma by proteomics. Proteomics..

[10] Moura AA, Souza CE, Stanley BA, Chapman DA, Killian GJ (2010). Proteomics of cauda epididymal fluid from mature Holstein bulls. J Proteome.

[11] Rego JP, Crisp JM, Moura AA, Nouwens AS, Li Y, Venus B (2014). Seminal plasma proteome of electroejaculated bos indicus bulls. Anim Reprod Sci.

[12] Lin Y, Liang A, He Y, Li Z, Li Z, Wang G, Sun F (2019). Proteomic analysis of seminal extracellular vesicle proteins involved in asthenozoospermia by iTRAQ. Mol Reprod Dev.

[13] Murdica V, Giacomini E, Alteri A, Bartolacci A, Cermisoni GC, Zarovni N (2019). Seminal plasma of men with severe asthenozoospermia contain exosomes that affect spermatozoa motility and capacitation. Fertil Steril.

[14] Simons M, Raposo G (2009). Exosomes--vesicular carriers for intercellular communication. Curr Opin Cell Biol.

[15] Guo H, Chang Z, Zhang Z, Zhao Y, Jiang X, Yu H, Zhang Y, Zhao R, He B (2019). Extracellular ATPs produced in seminal plasma exosomes regulate boar sperm motility and mitochondrial metabolism. Theriogenology..

[16] Du J, Shen J, Wang Y, Pan C, Pang W, Diao H, Dong W (2016). Boar seminal plasma exosomes maintain sperm function by infiltrating into the sperm membrane. Oncotarget..

[17] Koch S, Acebron SP, Herbst J, Hatiboglu G, Niehrs C (2015). Post-transcriptional Wnt signaling governs Epididymal sperm maturation. Cell..

[18] De Robertis EM, Ploper D (2015). Sperm motility requires Wnt/GSK3 stabilization of proteins. Dev Cell.

[19] Arienti G, Carlini E, Nicolucci A, Cosmi EV, Santi F, Palmerini CA (1999). The motility of human spermatozoa as influenced by prostasomes at various pH levels. Biol Cell.

[20] Vivacqua A, Siciliano L, Sabato M, Palma A, Carpino A (2004). Prostasomes as zinc ligands in human seminal plasma. Int J Androl.

[21] Aalberts M, Sostaric E, Wubbolts R, Wauben MW, Nolte-'t Hoen EN, Gadella BM (2013). Spermatozoa recruit prostasomes in response to capacitation induction. Biochim Biophys Acta.

[22] Gilany K, Minai-Tehrani A, Savadi-Shiraz E, Rezadoost H, Lakpour N (2015). Exploring the human seminal plasma proteome: an unexplored gold mine of biomarker for male infertility and male reproduction disorder. J Reprod Infertil.

[23] Utleg AG, Yi EC, Xie T, Shannon P, White JT, Goodlett DR (2003). Proteomic analysis of human prostasomes. Prostate..

[24] Poliakov A, Spilman M, Dokland T, Amling CL, Mobley JA (2009). Structural heterogeneity and protein composition of exosome-like vesicles (prostasomes) in human semen. Prostate..

[25] Yang C, Guo WB, Zhang WS, Bian J, Yang JK, Zhou QZ (2017). Comprehensive proteomics analysis of exosomes derived from human seminal plasma. Andrology..

[26] Suteevun T, Smith SL, Muenthaisong S, Yang X, Parnpai R, Tian XC (2006). Anomalous mRNA levels of chromatin remodeling genes in swamp buffalo (Bubalus bubalis) cloned embryos. Theriogenology..

[27] Brohi RD, Huo LJ (2017). Posttranslational modifications in spermatozoa and effects on male fertility and sperm viability. OMICS..

[28] Dixit S, Pandey V, Swain DK, Nigam R, Singh P (2016). Seminal plasma and sperm membrane proteins of buffalo and cattle bulls: A comparative study. Buffalo Bull.

[29] Fu Q, Pan L, Huang D, Wang Z, Hou Z, Zhang M (2019). Proteomic profiles of buffalo spermatozoa and seminal plasma. Theriogenology..

[30] Mathivanan S, Simpson RJ (2009). ExoCarta: a compendium of exosomal proteins and RNA. Proteomics..

[31] Mathivanan S, Fahner CJ, Reid GE, Simpson RJ (2012). ExoCarta 2012: database of exosomal proteins, RNA and lipids. Nucleic Acids Res.

[32] Keerthikumar S, Chisanga D, Ariyaratne D, Al Saffar H, Anand S, Zhao K (2016). ExoCarta: a web-based compendium of Exosomal cargo. J Mol Biol.

[33] Kalra H, Simpson RJ, Ji H, Aikawa E, Altevogt P, Askenase P (2012). Vesiclepedia: a compendium for extracellular vesicles with continuous community annotation. PLoS Biol.

[34] Vizcaino JA, Csordas A, Del-Toro N, Dianes JA, Griss J, Lavidas I (2016). 2016 update of the PRIDE database and its related tools. Nucleic Acids Res.

[35] Mathivanan S, Ji H, Simpson RJ (2010). Exosomes: extracellular organelles important in intercellular communication. J Proteome.

[36] Candenas L, Chianese R. Exosome composition and seminal plasma proteome: a promising source of biomarkers of male infertility. Int J Mol Sci. 2020;21(19):7022. 10.3390/ijms21197022. 10.3390/ijms21197022 PMC7583765 32987677

[37] Gangnuss S, Sutton-McDowall ML, Robertson SA, Armstrong DT (2004). Seminal plasma regulates corpora lutea macrophage populations during early pregnancy in mice. Biol Reprod.

[38] Cizmar P, Yuana Y (2017). Detection and characterization of extracellular vesicles by transmission and Cryo-transmission Electron microscopy. Methods Mol Biol.

[39] Li G, Marlin MC (2015). Rab family of GTPases. Methods Mol Biol.

[40] Shan MM, Sun SC (2021). The multiple roles of RAB GTPases in female and male meiosis. Hum Reprod Update.

[41] Dacheux JL, Dacheux F (2014). New insights into epididymal function in relation to sperm maturation. Reproduction..

[42] Fiedler SE, Dudiki T, Vijayaraghavan S, Carr DW (2013). Loss of R2D2 proteins ROPN1 and ROPN1L causes defects in murine sperm motility, phosphorylation, and fibrous sheath integrity. Biol Reprod.

[43] Chiriva-Internati M, Gagliano N, Donetti E, Costa F, Grizzi F, Franceschini B (2009). Sperm protein 17 is expressed in the sperm fibrous sheath. J Transl Med.

[44] Kierszenbaum AL, Tres LL, Rivkin E, Kang-Decker N, van Deursen JM (2004). The acroplaxome is the docking site of Golgi-derived myosin Va/Rab27a/b- containing proacrosomal vesicles in wild-type and Hrb mutant mouse spermatids. Biol Reprod.

[45] Garde J, Roldan ER (1996). Rab 3-peptide stimulates exocytosis of the ram sperm acrosome via interaction with cyclic AMP and phospholipase A2 metabolites. FEBS Lett.

[46] Eickhoff R, Baldauf C, Koyro HW, Wennemuth G, Suga Y, Seitz J (2004). Influence of macrophage migration inhibitory factor (MIF) on the zinc content and redox state of protein-bound sulphydryl groups in rat sperm: indications for a new role of MIF in sperm maturation. Mol Hum Reprod.

[47] Curry E, Safranski TJ, Pratt SL (2011). Differential expression of porcine sperm microRNAs and their association with sperm morphology and motility. Theriogenology..

[48] Sullivan R (2015). Epididymosomes: a heterogeneous population of microvesicles with multiple functions in sperm maturation and storage. Asian J Androl..

[49] Gibbs GM, Lo JC, Nixon B, Jamsai D, O'Connor AE, Rijal S (2010). Glioma pathogenesis-related 1-like 1 is testis enriched, dynamically modified, and redistributed during male germ cell maturation and has a potential role in sperm-oocyte binding. Endocrinology..

[50] Hurtado de Llera A, Martin-Hidalgo D, Gil MC, Garcia-Marin LJ, Bragado MJ (2012). AMP-activated kinase AMPK is expressed in boar spermatozoa and regulates motility. PLoS One.

[51] Bertoldo MJ, Faure M, Dupont J, Froment P (2015). AMPK: a master energy regulator for gonadal function. Front Neurosci.

[52] Hurtado de Llera A, Martin-Hidalgo D, Rodriguez-Gil JE, Gil MC, Garcia-Marin LJ, Bragado MJ (2013). AMP-activated kinase, AMPK, is involved in the maintenance of plasma membrane organization in boar spermatozoa. Biochim Biophys Acta.

[53] Zhu Z, Li R, Ma G, Bai W, Fan X, Lv Y (2018). 5′-AMP-activated protein kinase regulates goat sperm functions via energy metabolism in vitro. Cell Physiol Biochem.

[54] Peddinti D, Memili E, Burgess SC (2010). Proteomics-based systems biology modeling of bovine germinal vesicle stage oocyte and cumulus cell interaction. PLoS One.

[55] Inoue N, Ikawa M, Isotani A, Okabe M (2005). The immunoglobulin superfamily protein Izumo is required for sperm to fuse with eggs. Nature..

[56] Kanemori Y, Koga Y, Sudo M, Kang W, Kashiwabara S, Ikawa M (2016). Biogenesis of sperm acrosome is regulated by pre-mRNA alternative splicing of Acrbp in the mouse. Proc Natl Acad Sci U S A.

[57] Fujihara Y, Satouh Y, Inoue N, Isotani A, Ikawa M, Okabe M (2012). SPACA1-deficient male mice are infertile with abnormally shaped sperm heads reminiscent of globozoospermia. Development..

[58] Lin YN, Roy A, Yan W, Burns KH, Matzuk MM (2007). Loss of zona pellucida binding proteins in the acrosomal matrix disrupts acrosome biogenesis and sperm morphogenesis. Mol Cell Biol.

[59] Choi H, Jin S, Kwon JT, Kim J, Jeong J, Kim J (2016). Characterization of mammalian ADAM2 and its absence from human sperm. PLoS One.

[60] Lee S, Hong SH, Cho C (2020). Normal fertility in male mice lacking ADAM32 with testis-specific expression. Reprod Biol.

[61] Drabovich AP, Dimitromanolakis A, Saraon P, Soosaipillai A, Batruch I, Mullen B (2013). Differential diagnosis of azoospermia with proteomic biomarkers ECM1 and TEX101 quantified in seminal plasma. Sci Transl Med.

[62] Nagdas SK, McLean EL, Richardson LP, Raychoudhury S (2014). Identification and characterization of TEX101 in bovine Epididymal spermatozoa. Biochem Res Int.

[63] Yin L, Chung CM, Huo R, Liu H, Zhou C, Xu W (2009). A sperm GPI-anchored protein elicits sperm-cumulus cross-talk leading to the acrosome reaction. Cell Mol Life Sci.

[64] Li W, Guo XJ, Teng F, Hou XJ, Lv Z, Zhou SY (2013). Tex101 is essential for male fertility by affecting sperm migration into the oviduct in mice. J Mol Cell Biol.

[65] Fujihara Y, Tokuhiro K, Muro Y, Kondoh G, Araki Y, Ikawa M (2013). Expression of TEX101, regulated by ACE, is essential for the production of fertile mouse spermatozoa. Proc Natl Acad Sci U S A.

[66] Thomas K, Sung DY, Chen X, Thompson W, Chen YE, McCarrey J (2011). Developmental patterns of PPAR and RXR gene expression during spermatogenesis. Front Biosci (Elite Ed).

[67] Liu LL, Xian H, Cao JC, Zhang C, Zhang YH, Chen MM (2015). Peroxisome proliferator-activated receptor gamma signaling in human sperm physiology. Asian J Androl.

[68] Santoro M, Guido C, De Amicis F, Sisci D, Vizza D, Gervasi S (2013). Sperm metabolism in pigs: a role for peroxisome proliferator-activated receptor gamma (PPARgamma). J Exp Biol.

[69] Ishibashi K, Yamauchi K, Kageyama Y, Saito-Ohara F, Ikeuchi T, Marumo F (1998). Molecular characterization of human Aquaporin-7 gene and its chromosomal mapping. Biochim Biophys Acta.

[70] Saito K, Kageyama Y, Okada Y, Kawakami S, Kihara K, Ishibashi K (2004). Localization of aquaporin-7 in human testis and ejaculated sperm: possible involvement in maintenance of sperm quality. J Urol.

[71] Moretti E, Terzuoli G, Renieri T, Iacoponi F, Castellini C, Giordano C (2013). In vitro effect of gold and silver nanoparticles on human spermatozoa. Andrologia..

[72] Schuh K, Cartwright EJ, Jankevics E, Bundschu K, Liebermann J, Williams JC (2004). Plasma membrane Ca2+ ATPase 4 is required for sperm motility and male fertility. J Biol Chem.

[73] Patel R, Al-Dossary AA, Stabley DL, Barone C, Galileo DS, Strehler EE (2013). Plasma membrane Ca^2+^-ATPase 4 in murine epididymis: secretion of splice variants in the luminal fluid and a role in sperm maturation. Biol Reprod.

[74] Amann RP, Waberski D (2014). Computer-assisted sperm analysis (CASA): capabilities and potential developments. Theriogenology..

[75] Percie du Sert N, Ahluwalia A, Alam S, Avey MT, Baker M, Browne WJ (2020). Reporting animal research: explanation and elaboration for the ARRIVE guidelines 2.0. PLoS Biol.

[76] Momen-Heravi F (2017). Isolation of extracellular vesicles by ultracentrifugation. Methods Mol Biol.

[77] Gardiner C, Ferreira YJ, Dragovic RA, Redman CW, Sargent IL. Extracellular vesicle sizing and enumeration by nanoparticle tracking analysis. J Extracell Vesicles. 2013;2:19671. 10.3402/jev.v2i0.19671. 10.3402/jev.v2i0.19671 PMC3760643 24009893

[78] Lin Y, Xiong W, Xiao S, Li F, Lu Z, Yan J (2020). Pharmacoproteomics reveals the mechanism of Chinese dragon's blood in regulating the RSK/TSC2/mTOR/ribosome pathway in alleviation of DSS-induced acute ulcerative colitis. J Ethnopharmacol.

[79] Chen F, Fu Q, Pu L, Zhang P, Huang Y, Hou Z, Xu Z, Chen D, Huang F, Deng T, Liang X, Lu Y, Zhang M (2018). Integrated analysis of quantitative proteome and transcriptional profiles reveals the dynamic function of maternally expressed proteins after parthenogenetic activation of Buffalo oocyte. Mol Cell Proteomics.

[80] Dennis G, Sherman BT, Hosack DA, Yang J, Gao W, Lane HC (2003). DAVID: database for annotation, visualization, and integrated discovery. Genome Biol.

[81] Kanehisa M, Sato Y, Kawashima M, Furumichi M, Tanabe M (2016). KEGG as a reference resource for gene and protein annotation. Nucleic Acids Res.

[82] von Mering C, Huynen M, Jaeggi D, Schmidt S, Bork P, Snel B (2003). STRING: a database of predicted functional associations between proteins. Nucleic Acids Res.

[83] Egertson JD, MacLean B, Johnson R, Xuan Y, MacCoss MJ (2015). Multiplexed peptide analysis using data-independent acquisition and skyline. Nat Protoc.

