# Supplementary material for: Comparative proteomic analysis of seminal plasma exosomes in buffalo with high and low sperm motility
Source: BMC Genomics. 2023 Jan 9;24:8. doi: 10.1186/s12864-022-09106-2 (PMC9830767; doi:10.1186/s12864-022-09106-2)
Supplement: Supplementary file 1 — Additional file 1: Fig. S1. The full size image of western blots of Alix, TSG101 and CD81 in seminal plasma exosomes (SPEs), large extracellular vesicles (LEVs), and seminal plasma (SP) samples. Fig. S2. Functional classification based on Gene Ontology (GO) annotation of proteome profile in spermatozoa, seminal plasma (SP), and seminal plasma exosomes (SPEs). Fig. S3. Original image of protein-protein interaction (PPI) network of seminal plasma exosomes (SPEs). [file 12864_2022_9106_MOESM1_ESM.pdf]

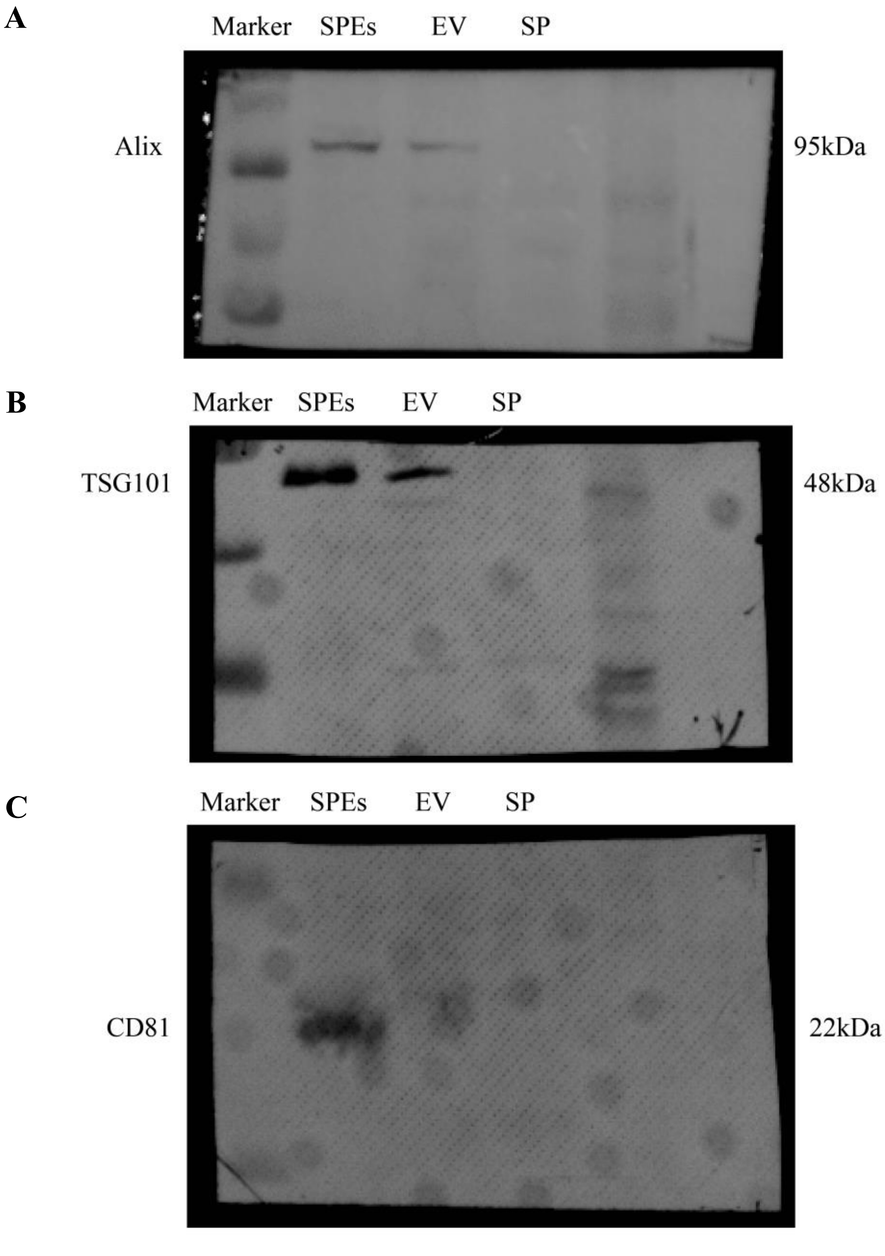

Fig. S1. The full size image of western blots of Alix, TSG101 and CD81 in seminal plasma exosomes (SPEs), large extracellular vesicles (LEVs), and seminal plasma (SP) samples. **A** Alix protein. **B** TSG101 protein. **C** CD81 protein

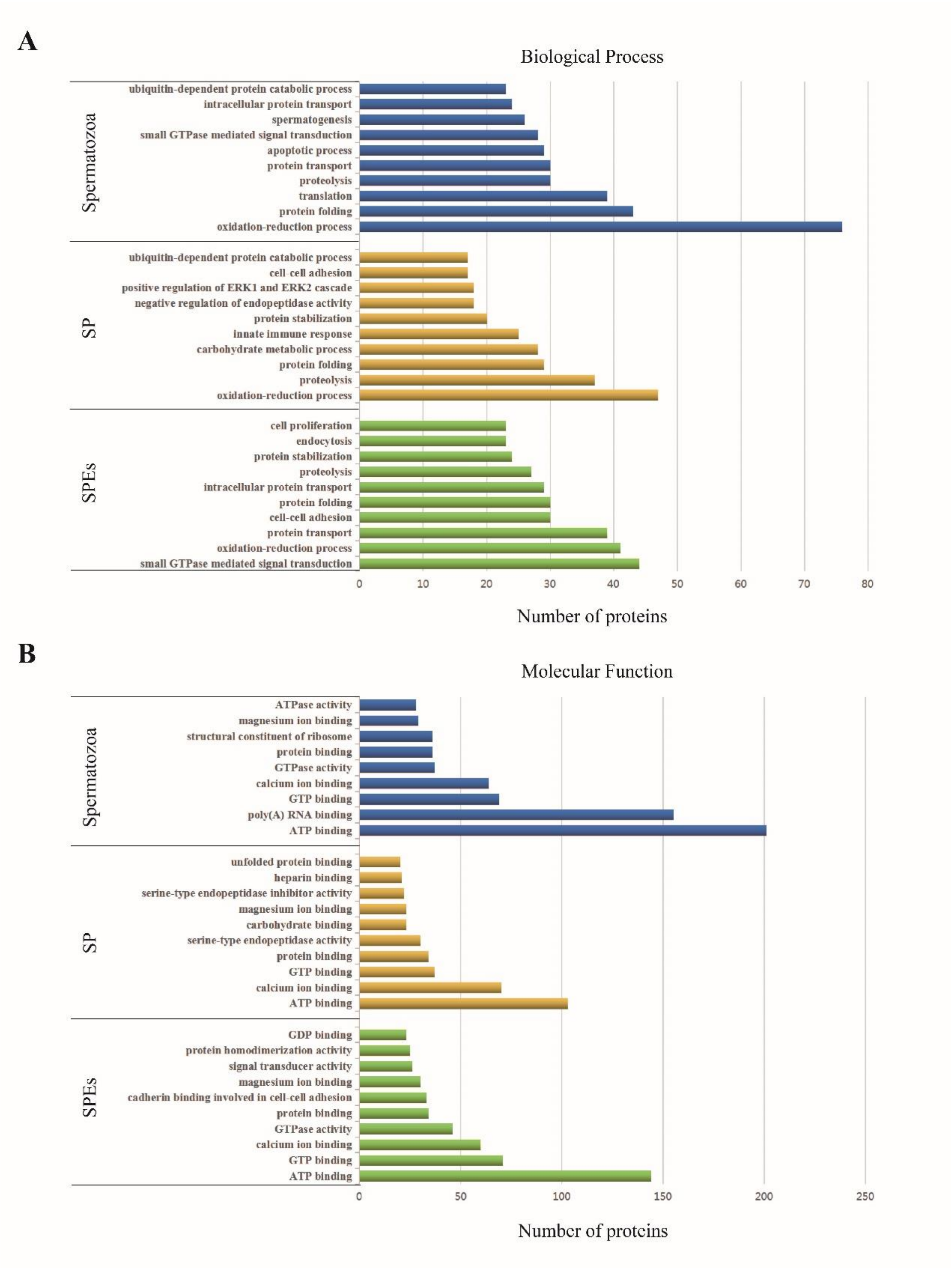

Fig. S2. Functional classification based on Gene Ontology (GO) annotation of proteome profile in spermatozoa, seminal plasma (SP), and seminal plasma exosomes (SPEs). **A** Functional classification of biological process. **B** Functional classification of molecular function

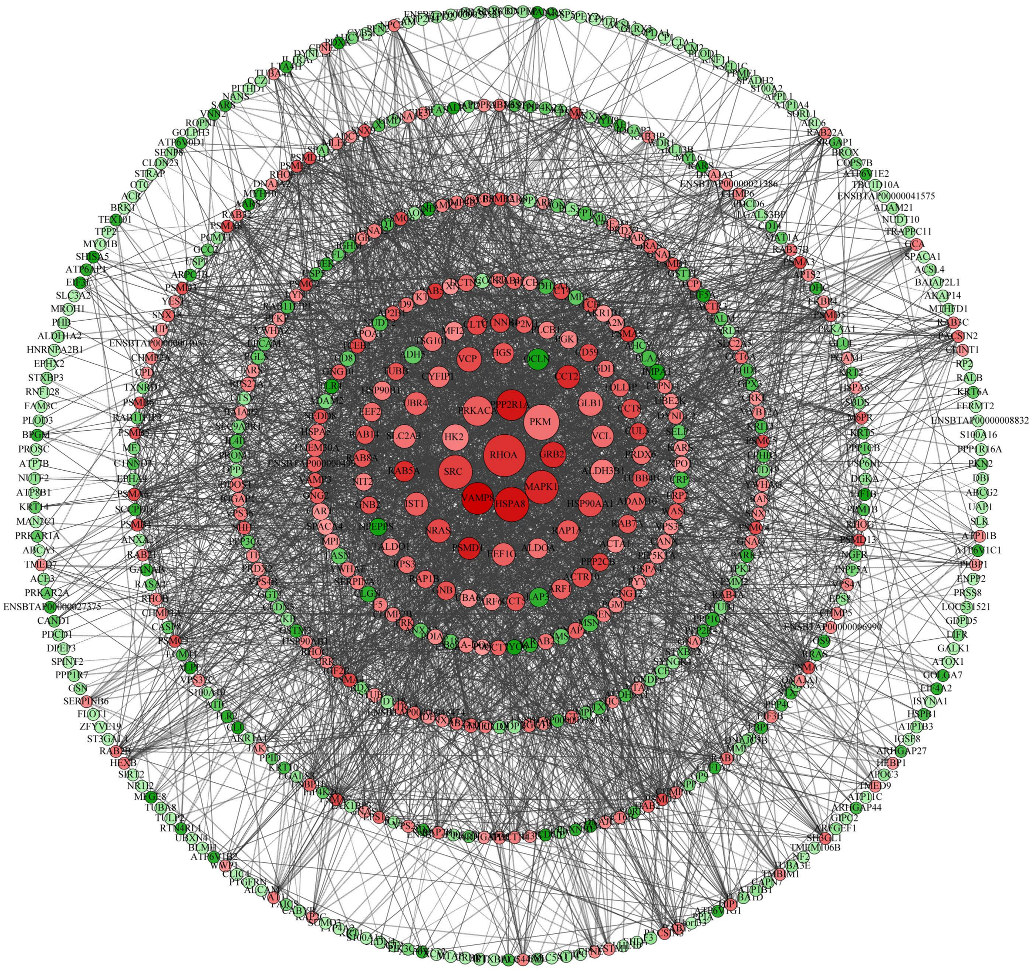

Fig. S3. Original image of protein-protein interaction (PPI) network of seminal plasma exosomes (SPEs)
